# Performance of model-based vs. permutation tests in the HEALing (Helping to End Addiction Long-term^SM^) Communities Study, a covariate-constrained cluster randomized trial

**DOI:** 10.1186/s13063-022-06708-9

**Published:** 2022-09-08

**Authors:** Xiaoyu Tang, Timothy Heeren, Philip M. Westgate, Daniel J. Feaster, Soledad A. Fernandez, Nathan Vandergrift, Debbie M. Cheng

**Affiliations:** 1 Department of Biostatistics, Boston University School of Public Health, 801 Massachusetts Avenue, Boston, MA 02219 USA; 2 Department of Biostatistics, University of Kentucky College of Public Health, Lexington, USA; 3 Department of Public Health Sciences, University of Miami, Coral Gables, FL USA; 4 Columbia University School of Social Work, New York, USA; 5 Department of Biomedical Informatics, The Ohio State University College of Medicine, Columbus, USA; 6 RTI International, Research Triangle, NC USA

**Keywords:** Covariate-constrained randomization, Model-based tests, Permutation tests, Cluster randomized trials, Negative binomial regression

## Abstract

**Background:**

The HEALing (Helping to End Addiction Long-term^SM^) Communities Study (HCS) is a multi-site parallel group cluster randomized wait-list comparison trial designed to evaluate the effect of the Communities That Heal (CTH) intervention compared to usual care on opioid overdose deaths. Covariate-constrained randomization (CCR) was applied to balance the community-level baseline covariates in the HCS. The purpose of this paper is to evaluate the performance of model-based tests and permutation tests in the HCS setting. We conducted a simulation study to evaluate type I error rates and power for model-based and permutation tests for the multi-site HCS as well as for a subgroup analysis of a single state (Massachusetts). We also investigated whether the maximum degree of imbalance in the CCR design has an impact on the performance of the tests.

**Methods:**

The primary outcome, the number of opioid overdose deaths, is count data assessed at the community level that will be analyzed using a negative binomial regression model. We conducted a simulation study to evaluate the type I error rates and power for 3 tests: (1) Wald-type *t*-test with small-sample corrected empirical standard error estimates, (2) Wald-type *z*-test with model-based standard error estimates, and (3) permutation test with test statistics calculated by the difference in average residuals for the two groups.

**Results:**

Our simulation results demonstrated that Wald-type *t*-tests with small-sample corrected empirical standard error estimates from the negative binomial regression model maintained proper type I error. Wald-type *z*-tests with model-based standard error estimates were anti-conservative. Permutation tests preserved type I error rates if the constrained space was not too small. For all tests, the power was high to detect the hypothesized 40% reduction in opioid overdose deaths for the intervention vs. comparison group both for the overall HCS and the subgroup analysis of Massachusetts (MA).

**Conclusions:**

Based on the results of our simulation study, the Wald-type *t*-test with small-sample corrected empirical standard error estimates from a negative binomial regression model is a valid and appropriate approach for analyzing cluster-level count data from the HEALing Communities Study.

**Trial registration:**

ClinicalTrials.gov http://www.clinicaltrials.gov; Identifier: NCT04111939

**Supplementary Information:**

The online version contains supplementary material available at 10.1186/s13063-022-06708-9.

## Background

Cluster randomized trials are widely used when researchers are aiming to study an intervention delivered at a group level (e.g., community, hospital, or school). However, techniques such as stratified randomization may not provide a sufficient balance of key baseline characteristics given randomization at the cluster level [1]. Balance of baseline covariates is important not only to improve power and precision [2], but also for the validity and credibility of studies as an imbalance of key covariates may bias results [3]. Many techniques exist to address the problem of covariate imbalance in cluster randomized trials, such as matching [4], stratification [5], and minimization [6]. Moulton proposed the covariate-constrained randomization (CCR) method which can balance multiple covariates simultaneously without the risk of over-stratification [7]. With CCR, all clusters are recruited prior to randomization and a set of key confounding variables is pre-specified along with the maximum degree of acceptable imbalance between randomized arms for each confounder. Among the set of group allocations that satisfy the pre-specified criteria for imbalance, i.e., the constrained randomization space, one is randomly selected to generate all randomized assignments for the trial. Covariate-constrained randomization generally does not include all possible allocations since it constrains the randomization space to those that meet the pre-specified degree of balance between randomized groups. Statistical analyses should match the study designs that generated the data, and thus, it is unclear whether test methods that do not account for the constrained space maintain proper type I and type II error rates.

Some cluster randomized trials may not have a sufficient number of clusters to ensure that large sample model-based inference is appropriate [8]. The permutation test is a method that does not require distributional assumptions and can be performed based on different test statistics [9]. Hence, it may be more appropriate than the model-based test in some settings. Fu et al. compared the performance of model-based and permutation tests from a generalized linear mixed model in cluster randomized trials that do not use CCR and found that the permutation test has the advantage of preserving nominal type I error for small studies and large intra-cluster correlation [10]. In terms of power, both methods had similar results. Murray demonstrated that if cluster randomization provides balance on confounders at baseline, type I error rate and power are similar for permutation tests and mixed-model regression [11].

Currently, there is limited research comparing permutation tests vs. model-based tests in the setting of cluster randomized trials that use CCR. Li et al. compared adjusted permutation tests and adjusted linear mixed effect model-based tests in the setting of CCR and concluded that in analyses adjusting for group-level covariates, both permutation tests and model-based tests can maintain the correct type I error rate as long as the number of possible allocations in the constrained randomization space is not too small [12]. Li et al. [13] compared permutation tests and model-based tests for randomized trials with binary outcomes in the setting of CCR. They found that when the prognostic group-level covariates are balanced by CCR at the design stage, both adjusted model-based tests (linearization *F*-test, KC-corrected GEE *t*-test) and adjusted permutation tests gain power and preserve test size compared to unadjusted analyses [13]. In both of the studies by Li, randomization is at the cluster level while the unit of analysis is at the individual level.

The purpose of this paper is to evaluate the performance of model-based tests and permutation tests in the covariate-constrained cluster randomized HEALing (Helping to End Addiction Long-term^SM^) Communities Study (HCS). The HCS is a multi-site parallel group cluster randomized wait-list comparison trial of the Communities That Heal (CTH) intervention [14–16]. The HCS was designed to evaluate the impact of the CTH intervention compared to usual care on opioid overdose deaths among 67 communities. The primary hypothesis is that communities in the intervention group will have a reduction in opioid-related deaths compared to those in the comparison group. This study was conducted in 4 states: Massachusetts, Kentucky, New York, and Ohio. CCR was used to balance 3 community-level baseline covariates: (1) rural/urban status, (2) population size, and (3) baseline opioid death rate. In the HCS, all key variables (i.e., outcomes and covariates) are aggregate community-level data. The primary outcome, the number of opioid overdose deaths, is count data assessed at the community level and analyzed using a negative binomial regression model [17].

As previous research on CCR focused on settings with individual-level data and did not analyze group-level count outcomes, it is unclear whether the results hold in the HCS setting. To address this question, we conducted a series of simulation studies to evaluate the performance of model-based tests and permutation tests in terms of type I error and power for the HCS setting. Our simulation studies are based on aggregate count data as the primary outcome of the HCS is the number of opioid overdose deaths at the community level. This setting is less common for cluster randomized trials, which often focus on individual-level data analyzed using models that control for within-cluster correlations [17]. The primary goal of this manuscript is to evaluate test performance for the HCS based on the pre-specified CCR constraints. The secondary objective is to investigate whether the number of clusters impacts test performance by assessing test performance for both the overall 4-site study as well as for a single state (i.e., Massachusetts only). We also explore the impact of the maximum degree of covariate imbalance by assessing results using alternative constraints for the CCR.

In the “Methods” section, we present the specific model-based and permutation tests we examined and describe our simulation methods. In the “Results” section, we present the results of the simulation study, summarizing the performance of the model-based tests and permutation tests in a range of scenarios. In the “Discussion” section, we discuss the results and limitations of our study.

## Methods

### Setting

The HCS was designed to evaluate the impact of the CTH intervention on opioid overdose deaths in 67 highly affected communities in Massachusetts, Kentucky, New York, and Ohio. Massachusetts, Kentucky, and New York each enrolled 16 communities and Ohio recruited 19 communities. The 67 communities were randomly assigned to either the intervention group during the first 2 years or a wait-list comparison group (continuing usual care) during the first 2 years using CCR stratified by state. CCR was used to ensure balance between intervention and comparison communities on three covariates at baseline. Specifically, the criteria were (1) less than a 0.2 standard deviation difference in the mean baseline opioid overdose death rate between intervention and comparison communities, (2) less than a 0.2 standard deviation difference in the overall mean population size between intervention and comparison communities, and (3) an equal number of rural and urban communities when there are an even number of communities and a difference of 1 otherwise. Based on these pre-specified constraints, the following were the number of acceptable allocations (total possible allocations): Massachusetts: 644 (12,870); Kentucky: 216 (12,870); New York: 650 (12,870); and Ohio: 6602 (92,378). The primary objective of the simulation study is to verify that the planned model-based test will maintain the proper type I error rate for the overall HCS and compare its performance to permutation tests that directly account for the CCR. Secondarily, we explore the performance of the model-based and permutation tests for a single site (i.e., Massachusetts) to assess whether a smaller number of clusters impacts test performance. In addition, we investigate the impact of the maximum degree of covariate imbalance on the performances of the tests for both the overall HCS and the single-site analysis of Massachusetts.

### Planned HCS analysis: negative binomial regression with small-sample correction

As noted above, the intervention group received the CTH intervention during the first 2 years of the study while the wait-list comparison group received usual care during the first 2 years of the study. The dependent variable *Y* is the number of opioid overdose deaths during year 2 of the trial. Data for the HCS study will be measured at the community level, and the planned primary analysis for the HCS study will use negative binomial regression as the dependent variable *Y* is count data. Negative binomial regression is a generalization of Poisson regression that does not require the assumption that the variance of the outcome count is equal to the mean. For the HCS, we expect over-dispersion in the data, i.e., *E*(*Y*) < *Var*(*Y*). As described by Walsh et al. and Westgate et al., the proposed negative binomial model will utilize small-sample adjusted empirical standard error estimates and degrees of freedom of the *t*-statistic equal to the number of communities minus the number of regression parameters [16, 17]. The negative binomial regression model that will be used to analyze the number of opioid overdose deaths is:

1 $${\mu}_i=E\left({Y}_i|X\right)=\exp \left(\log \left({n}_i\right)+{\beta}_0+{\beta}_{int}{X}_{i_{int ervention}}+{\beta}_{urban}{X}_{i_{urban}}+{\beta}_{dr}{X_i}_{deathrate}+{\beta}_{NY}{X}_{i_{NY}}+{\beta}_{KY}{X}_{i_{KY}}+{\beta}_{MA}{X}_{i_{MA}}\right)$$

where *i* indexes the individual community, baseline population *n*_*i*_ is the offset variable, *β*_0_ is the intercept, and *β*_*int*_, *β*_*urban*_, *β*_*dr*_, *β*_*NY*,_ *β*_*KY*,_ *β*_*MA*_ are corresponding regression coefficients for the intervention $\left({X}_{i_{intervention}}\right)$, rural/urban status $\left({X}_{i_{urban}}\right)$, baseline opioid death rate (*X*_*ideathrate*_), and state indicators for NY$\left({X}_{i_{NY}}\right)$, KY$\left({X}_{i_{KY}}\right)$, and MA$\left({X}_{i_{MA}}\right)$, respectively.

As noted by Murray et al. and Simon, the model used for analysis should match the design of the study in order to maintain an appropriate significance level [11, 18]. Hence, baseline population, rural/urban status, opioid death rate, and states are included in the model to improve efficiency as they were considered prognostic factors and included as covariates in the CCR. The dispersion parameter k satisfies $\mathit{\operatorname{var}}\left({Y}_i|{X}_i\right)={\mathsf{\mu}}_i+k{\mathsf{\mu}}_i^2$.

The small-sample corrected empirical standard error estimate that will be used in the primary HCS analyses follows the suggestion from Ford and Westgate, which is the average of the small-sample corrected empirical estimators proposed by Mancl and DeRouen, and Kauermann and Carroll. The Mancl and DeRouen and Kauermann and Carroll covariance estimators were both derived based on Taylor-series approximations (although they differ based on the assumptions made) and utilize leverage values [19, 20]. The Mancl and DeRouen estimator can be conservative, while the Kauermann and Carroll estimator can be liberal [19–21]. Ford and Westgate therefore proposed using the average of the two [21]. We provide details on how to obtain the estimator proposed by Ford and Westgate using R software in Supplementary Text 1.

### Tests evaluated in the simulation study

#### Model-based tests

We evaluated 2 model-based tests in our simulation study:

1. The first model-based test is from the planned primary analysis of the HCS described above, i.e., the Wald-type *t*-statistic utilizing small-sample corrected empirical standard errors (*SE*_*c*_) from the negative binomial regression model: $\frac{{\hat{\beta}}_{int}}{S{E_c}_{{\hat{\beta}}_{int}}}$. We assume that the *t*-statistic under null hypothesis follows a *t* distribution with degrees of freedom equal to *n* − *p* − 1, where *n* is the number of communities and *p* is the number of covariates. For HCS where *n* = 67 and *p*=6, the number of degrees of freedom is therefore 60.
2. The second model-based test is the Wald-type *z*-statistic from a standard negative binomial regression using model-based standard errors (SE): i.e.,$\frac{{\hat{\beta}}_{int}}{SE_{{\hat{\beta}}_{int}}}$. We assume that the Wald-type *z*-statistic under null hypothesis follows a standard normal distribution. We note that neither of the above model-based tests directly accounts for the constrained randomization space.

#### Permutation test

A permutation test is a statistical test in which the distribution of the test statistics under null hypothesis is obtained by calculating all possible values of the test statistic under all possible rearrangements of the study groups [22]. Permutation tests do not make assumptions about the sampling distribution of test statistics. We focused on the difference in the average residuals as the test statistic for our permutation test, an approach proposed by Gail et al. [23]. The residuals, *r*_*i*_, were defined as ${r}_i={Y}_i-{\hat{Y}}_i$. *Y*_*i*_ is the observed number of opioid overdose deaths during year 2 of the trial for community *i* and ${\hat{Y}}_i$ is the predicted follow-up number of opioid overdose deaths during year 2 of the trial for community *i*. To calculate the fitted value, ${\hat{Y}}_i,$ for community *i*, we fitted the negative binomial regression on rural/urban, baseline opioid overdose death rate for community *i*, and log of population size for community *i* as offset (note that intervention is not included in this model). We then obtained the average residual for the intervention group $\overline{r_1}={N}_1^{-1}\sum_{j=1}^{n_1}{r}_j$ where *N*_1_ is the sample size in the intervention group, and the average residual for the comparison group, i.e., $\overline{r_0}={N}_0^{-1}\sum_{j=1}^{n_0}{r}_j$ where *N*_0_is the sample size in the comparison group. Under the null hypothesis of no intervention effect, these residuals are independent of the intervention assignments. The resulting difference in average residuals test statistic is defined as U=$\overline{r_1}-$ $\overline{r_0}$. We calculated the average residual test statistics for each permutation of the treatment assignment in the permutation space to obtain the sampling distribution for the statistics. The space of the permutation test was determined based on the constrained randomization space of the HCS.

### Simulation study

We conducted a series of simulations to evaluate both type I error and power for the model-based and permutation tests. For type I error, we assumed that the true intervention effect is 0 and used a 2-sided *p*-value when evaluating type I error. For power, consistent with the design of the HCS, we assumed that the intervention group would experience a 40% reduction in opioid overdose deaths while the wait-list comparison group would experience no change. In addition to this scenario, we also assessed power for smaller effect sizes of 20% and 30%. For power, we report the probability of rejecting the null in the hypothesized direction when testing at the two-tailed 0.05 level. We ran 5000 iterations for the simulation. We note that with 5000 iterations, the precision of the simulation study, in terms of the resulting width of the 95% CI for the type I error (alpha=0.05), is $\pm 1.96\times \sqrt{\frac{0.05\left(1-0.05\right)}{5000}}=\pm 0.006$. For each iteration, we obtained a *p*-value corresponding to each model-based test and permutation test. We compared these *p*-values to the nominal level of 0.05 to determine the type I error and power for each test. The type I error and power were calculated conditionally on the particular randomized group allocation that was observed for the HCS.

#### Maximum degree of covariate imbalance in the CCR design

We investigated whether the maximum degree of covariate imbalance in the CCR design influenced the performance of tests by examining tighter constraints than those applied in the HCS, both for the overall HCS and within the single-site analysis of Massachusetts. We focused only on the constraints for continuous covariates, i.e., population size and opioid death rate, as the constraint for urban/rural status (i.e., equal for even numbers and differ by no more than 1 for odd numbers of communities) could not be further tightened. For population size and opioid death rate, we considered the following range of constraints: a difference between the 2 arms < 0.2 SD, 0.1 SD, and 0.05 SD of the overall mean. The allowable degree of covariate imbalance is directly related to the size of the constrained randomization space (i.e., number of acceptable allocations). The constraints we evaluated correspond to the following number of acceptable allocations for the overall HCS: 5.74 × 10^11^(0.2 SD), 6.23 × 10^7^ (0.1 SD), and 921,984 (0.05 SD), out of 1.96 × 10^17^ allocations in the unconstrained sampling space. We note that for the constraint with 0.05 SD, there is no acceptable allocation for Kentucky. Hence, we evaluated only the constraints with 0.2 SD and 0.1 SD for the overall HCS but included 0.05 SD in the single-site analysis of Massachusetts. For Massachusetts, the number of acceptable allocations corresponding to each constraint is as follows: 644 (.2 SD), 190 (.1 SD), and 44 (.05 SD), out of 12,870 possible allocations in the unconstrained sampling space.

#### Additional exploratory simulations

In order to explore the impact of the number of covariates and choice of allocations on the performance of different tests, we varied the number of covariates included in the regression models (i.e., we fit an unadjusted model, an adjusted model excluding urban/rural status, and an adjusted model excluding baseline death rate) as well as 3 new allocations randomly selected from the constrained space.

#### Data generation

To evaluate type I error and power, we simulated the primary outcome number of opioid overdose deaths using the following steps:

1. We first obtained the observed baseline number of opioid overdose deaths by averaging the number of deaths for the 2 years prior to the start of the study (2016 and 2017).
2. We then fitted a negative binomial regression model with the observed baseline number of opioid deaths from step 1 as the dependent variable, rural/urban status and state indicators as covariates, and the natural logarithm of population size for each community as the offset. After running the regression, we obtained the fitted value of the intercept *β*_0_ , the coefficient for rural/urban status *β*_*urban*_, coefficients for state indicators *β*_*NY*,_ *β*_*KY*,_ *β*_*MA*_, and the dispersion parameter *k*.
3. We simulated our outcome variable *Y*_*i*_ from a negative binomial distribution with mean calculated as $E\left({Y}_i|{X}_i\right)={\upmu}_i=\exp\ \Big(\log \left({n}_i\right)+{\beta}_0+{X}_{i_{intervention}}\ast \log \left(1- risk\ reduction\right)+{\beta}_{urban}{X}_{i_{urban}}+{\beta}_{NY}{X}_{i_{NY}}+{\beta}_{KY}{X}_{i_{KY}}+{\beta}_{MA}{X}_{i_{MA}}$and dispersion parameter *k*. When we evaluated the type I error rate, we assumed the true treatment effect is under the null hypothesis and thus the risk reduction is 0%. When we evaluated the power, the risk reduction was set at potential values under the alternative hypothesis: 20%, 30%, and 40%.
4. We used the random number generator in the R Software v3.6.2 (R Development Core Team, Vienna, Austria) to simulate 67 values from this distribution, one for each HCS community.

For analyses of the overall HCS, there are 67 communities across 4 states, resulting in a large number of possible allocations for permutation tests. To reduce the computational burden, within each of the 5000 iterations of the simulation, we randomly selected 3000 allocations from the constrained randomization space to estimate the sampling distribution for the permutation statistic.

For the subgroup analysis of Massachusetts, we simulated 16 values (one for each MA community) from the negative binomial distribution using the method above without state indicators. Because the actual observed HCS allocation in HCS satisfies the two constraints of 0.2 SD and 0.1 SD, we used the observed HCS allocation as the basis for testing for both of these constraints in order to control the effect of a random choice of allocation from the constrained space. For the tighter constraint (0.05 SD), which the observed MA allocation did not satisfy, we randomly selected an allocation from the constrained space. For the single-site analysis of Massachusetts, we estimated the sampling distribution for the permutation statistic using the all possible allocations from the constrained space.

## Results

**Fig. 1:**
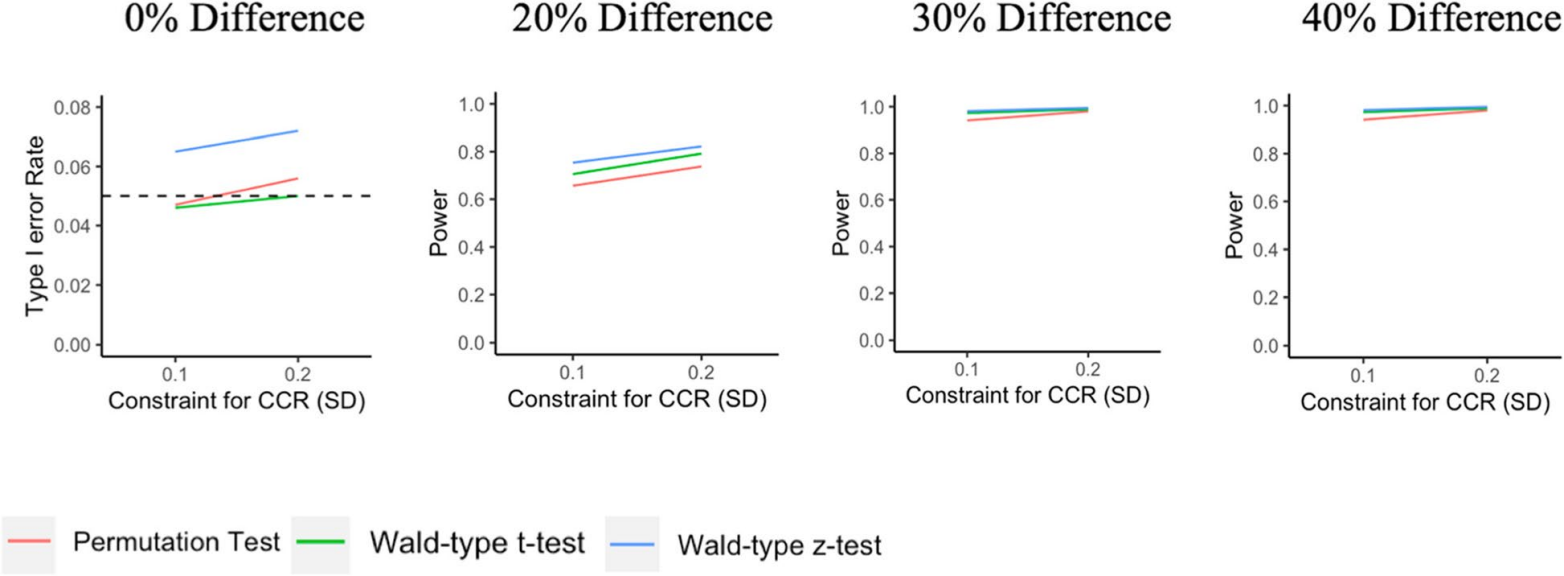
Type I error rate and power with varying maximum degrees of covariate imbalance for different tests for overall HCS

**Fig. 2:**
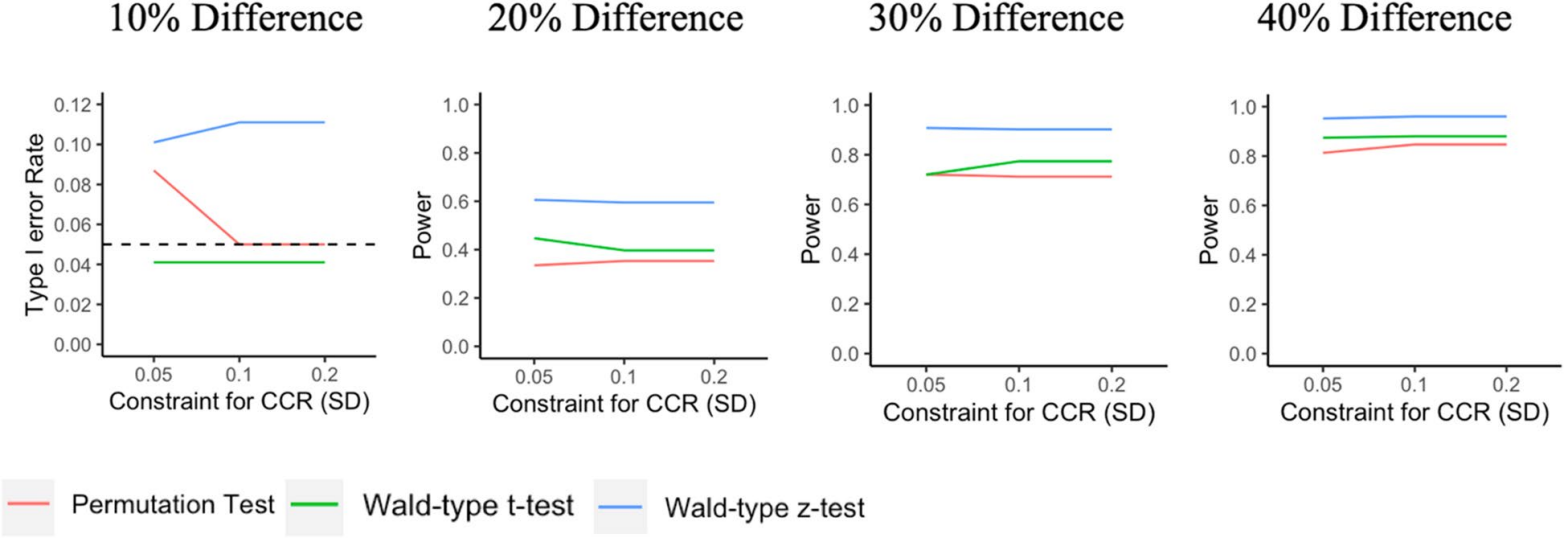
Type I error rate and power with varying maximum degrees of covariate imbalance for different tests for MA only

In the following, we present the type I error rate (Table 1) and power (Table 2) for the two model-based tests and the permutation test for the overall HCS and the single-site analysis of the Massachusetts subgroup, based on the CCR constraints used by the HCS (i.e., 0.2 SD difference for the continuous covariates population size and baseline opioid death rate). These results are also illustrated in Figs. 1 and 2.

**Table 1:** Type I error rate for the model-based and permutation tests for the HEALing Communities Study Design, overall (4 states) and for the subgroup analysis of Massachusetts

	Type I error	
Test type	Overall HCS	MA only
**Model-based tests**		
**Wald-type** ***t*-test**^a^	0.050	0.041
**Wald-type** ***z*-test**^b^	0.072	0.111
**Permutation test**		
**Difference in residuals**	0.056	0.050

The constraint used in the CCR is 0.2 SD for population size and baseline opioid death rate

^a^Small-sample corrected empirical standard error estimates

^b^Model-based standard error estimates

**Table 2:** Power for the model-based and permutation tests to detect various differences between groups in number of opioid overdose deaths

	Power					
	20% difference		30% difference		40% difference	
Test type	Overall HCS	MA only	Overall HCS	MA only	Overall HCS	MA only
**Model-based tests**						
**Wald-type** ***t*-test**^a^	0.792	0.397	0.989	0.774	1.000	0.880
**Wald-type** ***z*-test**^b^	0.822	0.595	0.995	0.902	1.000	0.960
**Permutation test**						
**Difference in residuals**	0.738	0.353	0.980	0.712	1.000	0.847

The constraint used in the CCR is 0.2 SD for population size and baseline opioid death rate

^a^Small-sample corrected empirical standard error estimates

^b^Model-based standard error estimates

Both the Wald-type *t*-test using small-sample corrected empirical standard error estimates and the permutation test based on the difference in residuals appear to preserve the type I error for both overall HCS and MA. However, the Wald-type *t*-test had higher power than the permutation test. The Wald-type *z*-test with model-based standard error estimates generally had the highest power; however, it appeared anti-conservative, with type I errors above 0.05 for both the overall HCS and MA subgroup analysis. Power for all three tests was high (i.e., >0.80), to detect differences of at least 30% between randomized groups in the overall HCS study and to detect a 40% difference for the MA analyses.

We also examined the impact of tighter constraints for covariate imbalance on both type I error (Table 3) and power (Table 4). For the overall HCS, we evaluated a 0.1 SD difference in population size and opioid death rate, and for the MA analysis, we evaluated constraints of 0.1 SD and 0.05 SD for both covariates. As before, Wald-type *t*-tests with small-sample corrected empirical standard error estimates preserved the type I error rates while the Wald-type *z*-tests with model-based standard error estimates were anti-conservative in terms of type I error rate. For the permutation tests, type I error rates appear to be preserved if the constraints used by the CCR are not too tight; otherwise, the number of possible allocations in the constrained space may be inadequate. This was observed for the scenario in the MA-only analyses with the degree of imbalance set at 0.05 SD, resulting in only 44 possible allocations. For a given effect size, the estimated power for each test did not change substantially with tighter constraints for either the overall HCS or the subgroup analysis of MA. The Wald-type *t*-tests had higher power than the permutation tests in nearly all scenarios.

**Table 3:** Type I error rates based on maximum degree of covariate imbalance

	Type I error		
	Overall HCS	MA only	MA only
**Max imbalance**^a^	**0.1SD**	**0.1SD**	**0.05SD**
**Model-based tests**			
**Wald-type** ***t*-test**^b^	0.046	0.041	0.041
**Wald-type** ***z*-test**^c^	0.065	0.111	0.101
**Permutation test**			
**Difference in residuals**	0.047	0.050	0.087

^a^Max imbalance on the constraints for population size and opioid death rate

^b^Small-sample corrected empirical standard error estimates

^c^Model-based standard error estimates

**Table 4:** Power based on the maximum degree of covariate imbalance

	20% difference			30% difference			40% difference		
	Overall HCS	MA only	MA only	Overall HCS	MA only	MA only	Overall HCS	MA only	MA only
**Max imbalance**^a^	**0.1SD**	**0.1SD**	**0.05SD**	**0.1SD**	**0.1SD**	**0.05SD**	**0.1SD**	**0.1SD**	**0.05SD**
**Model-based tests**									
**Wald-type** ***t*-test**^b^	0.706	0.397	0.447	0.973	0.774	0.720	0.999	0.880	0.874
**Wald-type** ***z*-test**^c^	0.754	0.595	0.606	0.981	0.902	0.908	0.999	0.960	0.952
**Permutation test**									
**Difference in residuals**	0.657	0.353	0.335	0.941	0.712	0.721	0.996	0.847	0.813

^a^Max imbalance on the constraints for population size and opioid death rate

^b^Small-sample corrected empirical standard error estimates

^c^Model-based standard error estimates

We report the distribution of the number of opioid overdose deaths from the various simulation scenarios in Supplementary Table 1. In additional exploratory analyses, we found that for the overall HCS, the type I error rates and the power did not have substantial changes across different scenarios, especially for the larger magnitudes of effect (Supplementary Tables 2, 3, 4, 5, 6 and 7). For the subgroup analysis of Massachusetts, when we decreased the number of covariates in the model, we observed that the type I error rates increased and the power decreased in nearly all the scenarios (Supplementary Tables 2, 3, 4, 5, 6 and 7). In addition, we found that the choice of allocation does not appear to influence our results (Supplementary Tables 8 and 9).

## Discussion

We examined the performance of model-based and permutation tests in terms of type I error rate and power under covariate-constrained randomization for the HEALing Communities Study (HCS). The HCS is a multi-site parallel group cluster randomized wait-list comparison trial of the Communities That Heal (CTH) intervention evaluating aggregate count outcomes at the community level (i.e., number of opioid overdose deaths). We assessed the performance of these tests for the primary analyses of the multi-site HCS as well as for a subgroup analysis of a single site, Massachusetts. Additionally, we explored the impact of implementing tighter covariate constraints for the CCR. We found that for both the overall HCS as well as the subgroup of MA, the primary analytic approach using Wald-type *t*-tests with small-sample corrected empirical standard error estimates from a negative binomial regression model maintained the proper type I error rate. The Wald-type *z*-tests with model-based standard error estimates, however, were anti-conservative. The permutation test based on the difference of average residuals preserved type I error rates when the degree of constraints was not too tight. Power was high for all tests to detect the hypothesized intervention effect of a 40% reduction in opioid deaths, both for the overall HCS and for the subgroup analysis of Massachusetts.

The key feature of the HCS study that differs from those included in previous research evaluating the performance of statistical tests in cluster randomized trials using CCR is the focus on an aggregate count outcome analyzed using a negative binomial regression model. Li et al. compared *F*-tests and permutation tests under CCR using linear mixed models [12] and Li et al. compared four model-based tests and two permutation tests [13] with binary outcomes. Consistent with both of these previous studies, we found that corrected model-based and permutation tests maintain the correct type I error rate and have similar power when the available allocations in the constrained randomization space are not too small. In the previous research by Li et al. noted above, *F*-tests with a linear mixed model, and linearization F-test and KC-corrected GEE *t*-test with binary outcomes preserved the type I error rates. In our analysis, the Wald-type *t*-tests with small-sample corrected empirical standard error estimates preserved the type I error rates but Wald-type *z*-tests with model-based standard error estimates did not, emphasizing the importance of the small-sample corrected empirical estimators when using model-based tests in cluster randomized trials with a small number of clusters. The correction factor we used was proposed by Ford and Westgate [21]. They found that by using the average of small-sample corrected empirical estimators proposed by Mancl and DeRouen, and Kauermann and Carroll [19–21, 24], nominal type I error rates can be consistently maintained, as confirmed by our simulation study. For permutation tests, Li et al. (2016), and Carter and Hood show that if the constrained randomization space contains larger than 100 possible allocations, permutation tests could maintain the desired type I errors and high power. Our findings show similar results in that when the constrained space contained >100 acceptable allocations, the type I error rates were preserved and sufficient power was achieved for the hypothesized 30% and 40% reductions in opioid deaths for the overall HCS, and for a 40% reduction in opioid deaths in the subgroup analysis of Massachusetts.

Our work has some limitations. First, our goal was to investigate the performance of permutation and model-based tests in the setting of HCS and the results may not be generalizable to other settings as we used community-level outcome data and HCS-specific baseline covariates. In addition, we used an empirical strategy to choose values for the impact of covariates, but did not examine the implications of varying the strength of those covariate relationships.

## Conclusions

Based on the results of our simulation study, the Wald-type *t*-test with small-sample corrected empirical standard error estimates from the negative binomial regression model is a valid and appropriate analytic approach for the HEALing Communities Study. It preserved the type I error rate and maintained high power for the hypothesized 40% reduction in opioid deaths for the overall multi-site analyses as well as for a subgroup analysis of a single site. In contrast, Wald-type *z*-tests with model-based standard error estimates resulted in inflated type I error rates. The permutation test based on the difference of average residuals appeared to maintain type I error in this setting. However, it had lower power compared to the Wald-type *t*-test with small-sample corrected empirical standard error estimates along with the added disadvantage of being computationally more complex.

## Supplementary Information

**Additional file 1: Supplementary Table 1.** Distribution of number of opioid overdose deaths from 5,000 simulations across 67 communities in the HCS*. **Supplementary Table 2.** Type I error rate for the Model-Based and Permutation Tests from the unadjusted model for the HEALing Communities Study Design, Overall (4 States) and for the subgroup analysis of Massachusetts. **Supplementary Table 3.** Power for the Model-Based and Permutation Tests from the unadjusted model to Detect Various Differences Between Groups in Number of Opioid Overdose Deaths. **Supplementary Table 4.** Type I error rate for the Model-Based and Permutation Tests from the fully adjusted model removing Urban/Rural as the covariate for the HEALing Communities Study Design, Overall (4 States) and for the subgroup analysis of Massachusetts. **Supplementary Table 5.** Power for the Model-Based and Permutation Tests from the fully adjusted model removing Urban/Rural as the covariate to Detect Various Differences Between Groups in Number of Opioid Overdose Deaths. **Supplementary Table 6.** Type I error rate for the Model-Based and Permutation Tests from the fully adjusted model removing baseline death rates as the covariate for the HEALing Communities Study Design, Overall (4 States) and for the subgroup analysis of Massachusetts. **Supplementary Table 7.** Power for the Model-Based and Permutation Tests from the fully adjusted model removing baseline death rates as the covariate to Detect Various Differences Between Groups in Number of Opioid Overdose Deaths. **Supplementary Table 8.** Type I error rate for the Model-Based and Permutation Tests for the HEALing Communities Study Design, Overall (4 States) and for the subgroup analysis of Massachusetts for different random allocations. **Supplementary Table 9.** Power for the Model-Based and Permutation Tests to Detect Various Differences Between Groups in Number of Opioid Overdose Deaths for different random allocations. **Supplementary Text 1.**

## Data Availability

The data that support the findings of this study are available from RTI International but restrictions apply to the availability of these data, which were used under license for the current study, and so are not publicly available. Data are however available from the authors upon reasonable request and with permission of RTI International.

## Abbreviations

HEAL: Helping to End Addiction Long-term^SM^
HCS: HEALing Communities Study
CTH: Communities That Heal
CCR: Covariate-constrained randomization
MA: Massachusetts
