# Supplementary material for: Performance of model-based vs. permutation tests in the HEALing (Helping to End Addiction Long-termSM) Communities Study, a covariate-constrained cluster randomized trial
Source: Trials. 2022 Sep 8;23:762. doi: 10.1186/s13063-022-06708-9 (PMC9461200; doi:10.1186/s13063-022-06708-9)
Supplement: Supplementary file 1 — Additional file 1: Supplementary Table 1. Distribution of number of opioid overdose deaths from 5,000 simulations across 67 communities in the HCS*. Supplementary Table 2. Type I error rate for the Model-Based and Permutation Tests from the unadjusted model for the HEALing Communities Study Design, Overall (4 States) and for the subgroup analysis of Massachusetts. Supplementary Table 3. Power for the Model-Based and Permutation Tests from the unadjusted model to Detect Various Differences Between Groups in Number of Opioid Overdose Deaths. Supplementary Table 4. Type I error rate for the Model-Based and Permutation Tests from the fully adjusted model removing Urban/Rural as the covariate for the HEALing Communities Study Design, Overall (4 States) and for the subgroup analysis of Massachusetts. Supplementary Table 5. Power for the Model-Based and Permutation Tests from the fully adjusted model removing Urban/Rural as the covariate to Detect Various Differences Between Groups in Number of Opioid Overdose Deaths. Supplementary Table 6. Type I error rate for the Model-Based and Permutation Tests from the fully adjusted model removing baseline death rates as the covariate for the HEALing Communities Study Design, Overall (4 States) and for the subgroup analysis of Massachusetts. Supplementary Table 7. Power for the Model-Based and Permutation Tests from the fully adjusted model removing baseline death rates as the covariate to Detect Various Differences Between Groups in Number of Opioid Overdose Deaths. Supplementary Table 8. Type I error rate for the Model-Based and Permutation Tests for the HEALing Communities Study Design, Overall (4 States) and for the subgroup analysis of Massachusetts for different random allocations. Supplementary Table 9. Power for the Model-Based and Permutation Tests to Detect Various Differences Between Groups in Number of Opioid Overdose Deaths for different random allocations. Supplementary Text 1. [file 13063_2022_6708_MOESM1_ESM.docx]

Supplementary Materials:

Supplementary Table 1. Distribution of number of opioid overdose deaths from 5,000 simulations across 67 communities in the HCS*.

|  | 0% difference | 20% Difference | 30% Difference | 40% Difference |
| --- | --- | --- | --- | --- |
| Overall HCS | | | | |
| Median | 3657 | 3252 | 3044 | 2854 |
| [Q1, Q3] | [3494, 3810] | [3100, 3390] | [2909, 3186] | [2719, 2976] |
| MA only | | | | |
| Median | 376.1 | 336.6 | 317.7 | 298.7 |
| [Q1, Q3] | [357, 395] | [319, 354] | [301, 335] | [282, 315] |

[Q1, Q3]: [25th percentile,75th percentile]

Note: The constraint used in the CCR is 0.2 SD for population size and baseline opioid death rate.

* Communities range in size from approximately 10,000 to 1,000,000 residents.

Supplementary Table 2. Type I error rate for the Model-Based and Permutation Tests from the unadjusted model for the HEALing Communities Study Design, Overall (4 States) and for the subgroup analysis of Massachusetts

|  | **Type I error** | |
| --- | --- | --- |
| **Test Type** | **Overall HCS** | **MA only** |
| **Model-Based Tests** |  | |
| **Wald-type t-test ^1^** | 0.046 | 0.054 |
| **Wald-type z-test ^2^** | 0.057 | 0.161 |
| **Permutation Test** |  | |
| **Difference in**  **Residuals** | 0.056 | 0.055 |

^1^Small-sample corrected empirical standard error estimates

^2^Model-based standard error estimates

Note: The constraint used in the CCR is 0.2 SD for population size and baseline opioid death rate.

Supplementary Table 3. Power for the Model-Based and Permutation Tests from the unadjusted model to Detect Various Differences Between Groups in Number of Opioid Overdose Deaths

|  | **Power** | | | | | |
| --- | --- | --- | --- | --- | --- | --- |
|  | **20% Difference** | | **30% Difference** | | **40% Difference** | |
| **Test Type** | **Overall HCS** | **MA only** | **Overall HCS** | **MA only** | **Overall HCS** | **MA only** |
| **Model-Based Tests** |  | | | |  | |
| **Wald-type t-test ^1^** | 0.783 | 0.350 | 0.994 | 0.649 | 1.000 | 0.840 |
| **Wald-type z-test ^2^** | 0.804 | 0.458 | 0.991 | 0.736 | 1.000 | 0.869 |
| **Permutation Test** |  | | | |  | |
| **Difference in**  **Residuals** | 0.757 | 0.310 | 0.983 | 0.675 | 1.000 | 0.879 |

Supplementary Table 4. Type I error rate for the Model-Based and Permutation Tests from the fully adjusted model removing Urban/Rural as the covariate for the HEALing Communities Study Design, Overall (4 States) and for the subgroup analysis of Massachusetts

|  | **Type I error** | |
| --- | --- | --- |
| **Test Type** | **Overall HCS** | **MA only** |
| **Model-Based Tests** |  | |
| **Wald-type t-test ^1^** | 0.047 | 0.049 |
| **Wald-type z-test ^2^** | 0.063 | 0.176 |
| **Permutation Test** |  | |
| **Difference in**  **Residuals** | 0.059 | 0.055 |

^1^Small-sample corrected empirical standard error estimates

^2^Model-based standard error estimates

Note: The constraint used in the CCR is 0.2 SD for population size and baseline opioid death rate.

Supplementary Table 5. Power for the Model-Based and Permutation Tests from the fully adjusted model removing Urban/Rural as the covariate to Detect Various Differences Between Groups in Number of Opioid Overdose Deaths

|  | **Power** | | | | | |
| --- | --- | --- | --- | --- | --- | --- |
|  | **20% Difference** | | **30% Difference** | | **40% Difference** | |
| **Test Type** | **Overall HCS** | **MA only** | **Overall HCS** | **MA only** | **Overall HCS** | **MA only** |
| **Model-Based Tests** |  | | | |  | |
| **Wald-type t-test ^1^** | 0.790 | 0.407 | 0.990 | 0.732 | 1.000 | 0.826 |
| **Wald-type z-test ^2^** | 0.826 | 0.591 | 0.994 | 0.856 | 1.000 | 0.924 |
| **Permutation Test** |  | | | |  | |
| **Difference in**  **Residuals** | 0.758 | 0.359 | 0.984 | 0.671 | 1.000 | 0.817 |

Supplementary Table 6. Type I error rate for the Model-Based and Permutation Tests from the fully adjusted model removing baseline death rates as the covariate for the HEALing Communities Study Design, Overall (4 States) and for the subgroup analysis of Massachusetts

|  | **Type I error** | |
| --- | --- | --- |
| **Test Type** | **Overall HCS** | **MA only** |
| **Model-Based Tests** |  | |
| **Wald-type t-test ^1^** | 0.056 | 0.055 |
| **Wald-type z-test ^2^** | 0.073 | 0.173 |
| **Permutation Test** |  | |
| **Difference in**  **Residuals** | 0.056 | 0.053 |

^1^Small-sample corrected empirical standard error estimates

^2^Model-based standard error estimates

Note: The constraint used in the CCR is 0.2 SD for population size and baseline opioid death rate.

Supplementary Table 7. Power for the Model-Based and Permutation Tests from the fully adjusted model removing baseline death rates as the covariate to Detect Various Differences Between Groups in Number of Opioid Overdose Deaths

|  | **Power** | | | | | |
| --- | --- | --- | --- | --- | --- | --- |
|  | **20% Difference** | | **30% Difference** | | **40% Difference** | |
| **Test Type** | **Overall HCS** | **MA only** | **Overall HCS** | **MA only** | **Overall HCS** | **MA only** |
| **Model-Based Tests** |  | | | |  | |
| **Wald-type t-test ^1^** | 0.767 | 0.377 | 0.983 | 0.683 | 1.000 | 0.802 |
| **Wald-type z-test ^2^** | 0.807 | 0.500 | 0.995 | 0.795 | 1.000 | 0.858 |
| **Permutation Test** |  | | | |  | |
| **Difference in**  **Residuals** | 0.725 | 0.327 | 0.981 | 0.687 | 1.000 | 0.811 |

**Results of using other allocations in the constrained space:**

Supplementary Table 8. Type I error rate for the Model-Based and Permutation Tests for the HEALing Communities Study Design, Overall (4 States) and for the subgroup analysis of Massachusetts for different random allocations

|  | **Type I error** | |
| --- | --- | --- |
| **Test Type** | **Overall HCS** | **MA only** |
| **Allocation 1** | | |
| **Model-Based Tests** |  | |
| **Wald-type t-test ^1^** | 0.051 | 0.049 |
| **Wald-type z-test ^2^** | 0.074 | 0.113 |
| **Permutation Test** |  | |
| **Difference in**  **Residuals** | 0.057 | 0.053 |
| **Allocation 2** | | |
| **Model-Based Tests** |  |  |
| **Wald-type t-test ^1^** | 0.048 | 0.045 |
| **Wald-type z-test ^2^** | 0.076 | 0.107 |
| **Permutation Test** |  |  |
| **Difference in**  **Residuals** | 0.052 | 0.048 |
| **Allocation 3** | | |
| **Model-Based Tests** |  |  |
| **Wald-type t-test ^1^** | 0.050 | 0.041 |
| **Wald-type z-test ^2^** | 0.072 | 0.111 |
| **Permutation Test** |  |  |
| **Difference in**  **Residuals** | 0.056 | 0.050 |

^1^Small-sample corrected empirical standard error estimates

^2^Model-based standard error estimates

Note: The constraint used in the CCR is 0.2 SD for population size and baseline opioid death rate.

Supplementary Table 9. Power for the Model-Based and Permutation Tests to Detect Various Differences Between Groups in Number of Opioid Overdose Deaths for different random allocations

|  | **Power** | | | | | |
| --- | --- | --- | --- | --- | --- | --- |
|  | **20% Difference** | | **30% Difference** | | **40% Difference** | |
| **Test Type** | **Overall HCS** | **MA only** | **Overall HCS** | **MA only** | **Overall HCS** | **MA only** |
| **Allocation 1** | | | | | | |
| **Model-Based Tests** |  | | | |  | |
| **Wald-type t-test ^1^** | 0.788 | 0.330 | 0.978 | 0.722 | 1.000 | 0.862 |
| **Wald-type z-test ^2^** | 0.810 | 0.542 | 0.997 | 0.894 | 1.000 | 0.920 |
| **Permutation Test** |  | | | |  | |
| **Difference in**  **Residuals** | 0.726 | 0.319 | 0.972 | 0.673 | 1.000 | 0.800 |
| **Allocation 2** | | | | | | |
| **Model-Based Tests** |  |  |  |  |  |  |
| **Wald-type t-test ^1^** | 0.800 | 0.387 | 0.966 | 0.787 | 1.000 | 0.889 |
| **Wald-type z-test ^2^** | 0.842 | 0.580 | 0.989 | 0.912 | 1.000 | 0.970 |
| **Permutation Test** |  |  |  |  |  |  |
| **Difference in**  **Residuals** | 0.767 | 0.363 | 0.979 | 0.732 | 1.000 | 0.867 |
| **Allocation 3** | | | | | | |
| **Model-Based Tests** |  |  |  |  |  |  |
| **Wald-type t-test ^1^** | 0.808 | 0.400 | 0.978 | 0.789 | 1.000 | 0.899 |
| **Wald-type z-test ^2^** | 0.843 | 0.589 | 0.998 | 0.900 | 1.000 | 0.979 |
| **Permutation Test** |  |  |  |  |  |  |
| **Difference in**  **Residuals** | 0.756 | 0.342 | 0.986 | 0.740 | 1.000 | 0.850 |

^1^Small-sample corrected empirical standard error estimates

^2^Model-based standard error estimates

Note: The constraint used in the CCR is 0.2 SD for population size and baseline opioid death rate.

Supplementary Text 1

To obtain the small-sample correction proposed by Ford and Westgate, we use the direct average of the small-sample corrected empirical estimators proposed by Mancl and DeRouen, and Kauermann and Carroll.[1]

The Mancl and DeRouen covariance estimator intends to reduce the bias of the residual estimator and uses the expectation of residuals estimator in covariance calculation.[2]. This estimator can be calculated using R software (version 3.6.2) using the vcovCL() function from the sandwich package with type=”HC3”.

The Kauermann and Carroll estimator uses the leverage-adjusted residual to correct the downward bias of the empirical sandwich estimator.[3] This estimator can be calculated using using R software (version 3.6.3) the vcovCL() function from the sandwich package with type=”HC2”.

Mancl and DeRouen, and Kauermann and Carroll both proposed the bias corrections for the empirical sandwich estimator, but they are slightly conservative and liberal, respectively.[1-3] Ford and Westgate proposed to use the direct average of the small-sample corrected empirical estimators proposed by Mancl and DeRouen, and Kauermann and Carroll.[1] Sample R code to calculate this estimator is provided in the following:

nbresult<-glm.nb(count~Urban_Rural+deathrate+arm++stateNY+stateKY+stateMA+offset(log(Population)),data=MH)

sumnb<-coeftest(nbresult,vcov= vcovHC(nbresult,type="HC3"),df=67-6-1) # using the Mancl and DeRouen estimator

sumnb2<-coeftest(nbresult,vcov= vcovHC(nbresult,type="HC2"),df=67-6-1) # using the Kauermann and Carroll estimator

#df is the degrees of freedom, equal to number of communities-number of covarites-1

se<-(sumnb[[8]]+sumnb2[[8]])/2 #average of the Mancl and DeRouen, and Kauermann and Carroll estimators.

#the index may change with the position of covariates and different number of covariates included. Users and look at the sumnb and sumbn2 to find the correct index.

**References**

1. Ford, W.P. and P.M. Westgate, *Improved standard error estimator for maintaining the validity of inference in cluster randomized trials with a small number of clusters.* Biom J, 2017. **59**(3): p. 478-495.

2. Mancl, L.A. and T.A. DeRouen, *A covariance estimator for GEE with improved small-sample properties.* Biometrics, 2001. **57**(1): p. 126-34.

3. Kauermann, G. and R. Carrol, *A note on the efficiency of sandwich covariance matrix estimation.* Journal of the American Statistical Association 2001. **96**: p. 1387-1396.
